# Label-Free Split Aptamer Sensor for Femtomolar Detection of Dopamine by Means of Flexible Organic Electrochemical Transistors

**DOI:** 10.3390/ma13112577

**Published:** 2020-06-05

**Authors:** Yuanying Liang, Ting Guo, Lei Zhou, Andreas Offenhäusser, Dirk Mayer

**Affiliations:** Institute of Biological Information Processing, JARA-Fundamentals of Future Information Technology, Forschungszentrum Jülich, 52425 Jülich, Germany; liangyuanying1@hotmail.com (Y.L.); guoting0615@163.com (T.G.); le.zhou@fz-juelich.de (L.Z.); a.offenhaeusser@fz-juelich.de (A.O.)

**Keywords:** aptamer, flexible organic electrochemical transistors, dopamine, femtomolar sensitivity, biosensor

## Abstract

The detection of chemical messenger molecules, such as neurotransmitters in nervous systems, demands high sensitivity to measure small variations, selectivity to eliminate interferences from analogues, and compliant devices to be minimally invasive to soft tissue. Here, an organic electrochemical transistor (OECT) embedded in a flexible polyimide substrate is utilized as transducer to realize a highly sensitive dopamine aptasensor. A split aptamer is tethered to a gold gate electrode and the analyte binding can be detected optionally either via an amperometric or a potentiometric transducer principle. The amperometric sensor can detect dopamine with a limit of detection of 1 μM, while the novel flexible OECT-based biosensor exhibits an ultralow detection limit down to the concentration of 0.5 fM, which is lower than all previously reported electrochemical sensors for dopamine detection. The low detection limit can be attributed to the intrinsic amplification properties of OECTs. Furthermore, a significant response to dopamine inputs among interfering analogues hallmarks the selective detection capabilities of this sensor. The high sensitivity and selectivity, as well as the flexible properties of the OECT-based aptasensor, are promising features for their integration in neuronal probes for the in vitro or in vivo detection of neurochemical signals.

## 1. Introduction

The ability to detect small-molecule neurotransmitters is crucial for understanding neuronal information processing, related neurochemical processes, and brain functions in general [1]. Dopamine, as one of the most important neurotransmitters of the human central nervous system, is involved in the regulation of many behavioral responses and brain functions [2], and abnormal levels are symptomatic for several neuronal diseases, such as Parkinson’s, Alzheimer’s, Tourette’s syndrome, and schizophrenia [3]. Therefore, the effective detection of dopamine in biological systems is essential for disease identification and subsequent adequate treatment. Several methods have been developed for monitoring or detecting this analyte, including electrochemical [4,5,6,7,8,9] ultraviolet–visible spectroscopy [10], mass spectroscopy [11], and liquid chromatography [12]. Among them, electrochemical approaches are versatile and promising due to their low fabrication costs, fast response, high sensitivity, and their easy miniaturization [13,14,15]. Aptamer, as one of the target recognition components of electrochemical sensors, has attracted plenty of attention because of its high selectivity, flexibility, high affinity [16], low cost, and easy fabrication [17]. However, due to the variable distribution of dopamine concentrations in different body fluids and tissues (in the range of fM [18] to μM [19]), as well as the interference from some analogues or ascorbic acid and uric acid, which share similar oxidation potentials with dopamine [20], it is challenging for conventional electrochemical methods to selectively detect dopamine at low concentrations.

Organic electrochemical transistors (OECTs) have emerged as robust alternatives to state-of-the-art sensors, since they were introduced by White et al. in 1984 [21], due to their intrinsically amplifying characteristics [22], biocompatibility, ease of fabrication, fast switching speed compared to standard electrolyte-gated organic field effect transistors [23], and their operation in aqueous solutions as an ion-to-electron converter [24]. Previously reported work has appreciated OECTs as an excellent platform for the label-free and high-sensitivity detection of a wide range of targets, ranging from proteins [25], cells [26], DNA [27], and glucose [28], etc. For example, a previous work used aptamer-modified gate electrodes for the selective detection of an ATP target with extremely low detection limits, which was four orders of magnitude lower than that of the corresponding amperometric aptasensor [24]. Liao et al. developed OECT-based dopamine sensors with Nafion or chitosan-modified Pt gate electrodes, which showed a low detection limit (5 nM) and could effectively exclude interference from uric acid and ascorbic acid [29]. Tang et al. compared the sensitivity of OECTs for the detection of dopamine by using different gate electrodes and found that Pt enabled the lowest detection limit of 5 nM [30]. However, dopamine detection methods that are capable of simultaneously demonstrating high sensitivity, selectivity, and a wide detection range are still lacking for clinical application.

**Scheme 1:**
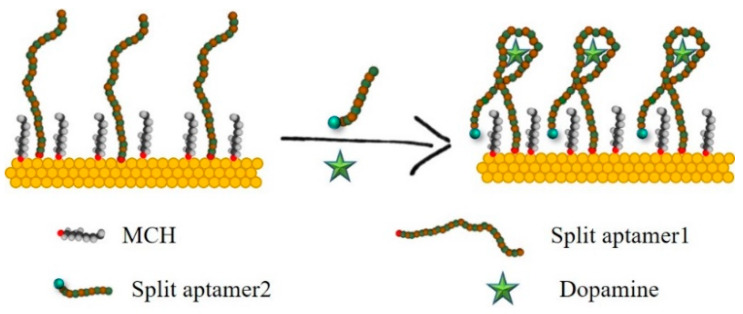
Schematic representation of the developed amperometric aptasensor for dopamine detection based on the structural assembly using split aptamers labeled with a methylene blue redox group (MB, green circle, split aptamer2) and surface tethered anchor strands (split aptamer1). A gold electrode is modified by the label-free aptamer1 and backfilled with blocking molecules MCH. A charge transfer occurs as a consequence of a molecular recognition event, resulting in the formation of an intact folded aptamer1/dopamine/aptamer2 sandwich structure.

In the present work, we embedded interdigitated OECTs (iOECTs) in a flexible polyimide substrate and utilized aptamer-modified gold electrodes as gate electrodes. Split aptamers were employed to get rid of the electrochemical response of the blank sample, in which one receptor fragment (aptamer1) is covalently attached to the surface of a gold electrode, as described in our previous work [31], and the other fragment (aptamer2) is used for signaling, labeled with a redox group (methylene blue) at the distal end, as shown in Scheme 1. Once the analyte dopamine is administered, the target can, on the one hand, induce the association of the two aptamer fragments into an aptamer1/dopamine/aptamer 2 sandwich structure, which may increase the concentration of the redox probe at the electrode surface, and on the other hand, decrease the distance between the gold electrode and the redox group, which therefore facilitates the charge transfer and generates a detectable electrochemical signal via an amperometric transducer principle [32].

The same gold electrode can be also utilized as a gate in the potentiometric iOECT transducer system and therefore shares the same recognition process as the amperometric transducer principle. However, the iOECT transducer relies on a change in the gate potential caused by the binding events between aptamer2/target and aptamer1 [24]. We found that the flexible iOECT-based aptasensor exhibited an ultralow detection limit of 0.5 fM, which is lower than that of the corresponding amperometric sensor and all previously reported electrochemical sensors for dopamine detection. At the same time, it conserves the high selectivity over analogues and its regeneration performance. 

## 2. Materials and Methods

### 2.1. Reagents

Dopaminehydrochloride (DA) and its analogues, as well as the chromium etchant, were purchased from Sigma (Sigma-Aldrich Chemie GmbH, Munich, Germany). Epoxy (302-3M, John P. Kummer GmbH, Augsbur, Germany) and polydimethylsiloxane (PDMS, Dow Corning Corporation, Wiesbaden, Germany) were used for the encapsulation of the flexible chip, which was described in detail in our previous work [24,26]. Two split-dopamine aptamers with the sequences of

Aptamer 1: TTC GCA GGT GTG GAG TGA CGT CG-(CH2)6-SH
Aptamer 2: MB-(CH2)6-CGA CGC CAG TTT GAA GGT TCG

were purchased from FRIZ Biochem (Neuried, Germany). Ten micromolar TE buffer (pH 8.0) was used to prepare the stock solutions of both DNA probes, whose concentrations were separately determined by using UV–vis spectroscopy to obtain the average absorbances value at 260 nm.

### 2.2. Fabrication Processes Flexible Organic Electrochemical Transistors

Flexible OECTs were fabricated according to the previously reported methods [26], including the deposition of a Cr/Au/Cr layer, metal, and Poly(3,4-ethylenedioxythiophene) doped with poly(styrenesulfonate) (PEDOT:PSS) (Clevios PH1000, Heraeus Clevios GmbH, Leverkusen, Germany). The PEDOT:PSS channel, consisting of 10% (*v*/*v*) dimethyl sulfoxide and 1% (*v*/*v*) 3-glycidoxypropyltrimethoxysilane with a channel area (polymer specifically between both electrodes) of 30 μm × 22 μm were utilized, which had similar dimensions as individual cardiomyocyte-like HL-1 cells and could record action potentials from electrogenic cells [33].

### 2.3. Stepwise Preparation of Aptamer-Based Sensors

An Au macroelectrode (ME), which was used as the gate electrode for the iOECTs, was firstly annealed with a hydrogen flame for ~10 s to remove the organic contaminations from the surface, followed by immersing it into ethanol and Milli-Q water for further cleaning. Oxidation and reduction scans were performed in a 50 mM H_2_SO_4_ solution over the potential range of −0.15 V to 1.55 V with a scan rate of 1 V/s and a step width of 0.01 V for final electrochemical annealing and determining its surface area (a scan rate of 0.1 V/s) [24,34]. Prior to incubation, the aptamer1 stock solution was mixed with 10 mM Tris-(2-carboxyethyl) phosphine hydrochloride (TCEP) (Sigma-Aldrich Chemie GmbH, Munich, Germany) for 1 h to reduce the disulfide bonds between the aptamer1 molecules. The clean Au macroelectrode surface was incubated with 0.5 μM aptamer1 in 10 mM high-salt Tris buffer (Tris, 1.5 M NaCl, pH = 7.4) overnight. A self-assembled monolayer was formed by thiol–gold bonding between the aptamer1 molecules and the gold electrode surface. The modified electrode was rinsed gently, first with 10 mM Tris buffer three times and consecutively with ethanol another three times to eliminate the non-bonded aptamer. Afterwards, the electrode was immersed in 1 mM 6-mercapto-1-hexanol (MCH)/ethanol solution for 1 h to completely block the electrode surface, which was then rinsed using the abovementioned method, but in the reverse order. For the regeneration measurement, 2 M NaCl solution was used by immersing the aptamer-modified electrode in the solution for 5 min to release the aptamer2 and analyte molecules, followed by rinsing with 10 mM Tris buffer to remove the residues.

### 2.4. Electrochemical Characterization

A conventional three-electrode setup was used for the electrochemical characterization of the amperometric sensors, including a platinum wire coil as a counter electrode, a micro Ag/AgCl electrode saturated (Micro DRIREF-450, World precision instruments, Sarasota, FL, USA) as a reference electrode, and the modified gold electrodes described above as working electrodes. Cyclic voltammetry and square wave voltammetry (SWV) were recorded by an Autolab potentiostat PGSTAT302 (Eco Chemie, Utrech, Netherland) in a 0.5 μM aptamer2 solution dissolved in 10 mM Tris buffer. The potential for SWV measurement varied from −0.6 V to 0.2 V (vs. Ag/AgCl) at a selected AC frequency (5 Hz, 10 Hz, 30 Hz, 50 Hz, 70 Hz, and 90 Hz). Dopamine hydrochloride powder (Sigma-Aldrich Chemie GmbH, Munich, Germany) was firstly dissolved in 10 mM Tris buffer and then added to the 0.5 μM aptamer2 solution to obtain different concentrations of dopamine. Subsequently, the aptamer1-modified gold electrode was incubated in the electrolyte for 10 min, which showed the highest current response to 10 μM dopamine (Figure S1), followed by rinsing with Tris buffer three times. Similar approaches were applied for the investigations of cross-sensitivities to interfering molecules, such as ascorbic acid (AA) and uric acid (UA), as well as other common neurotransmitters, such as gamma-aminobutyric acid (GABA) and glutamic acid (Glu).

### 2.5. Characterization of Tranfer Properties of iOECTs

The measurements of the output characteristics of the flexible iOECTs and the transfer characteristics for the detection of dopamine targets were performed using a Keithley 4200 semiconductor analyzer (Tektronix, Munich, Germany) [24]. Furthermore, the flexible iOECT-based aptasensor was operated in an open cell system [24]. All data points and error bars representing the average signals and standard deviations for dopamine detection were obtained from at least three independent flexible iOECTs. 

## 3. Results and Discussion

**Figure 1:**
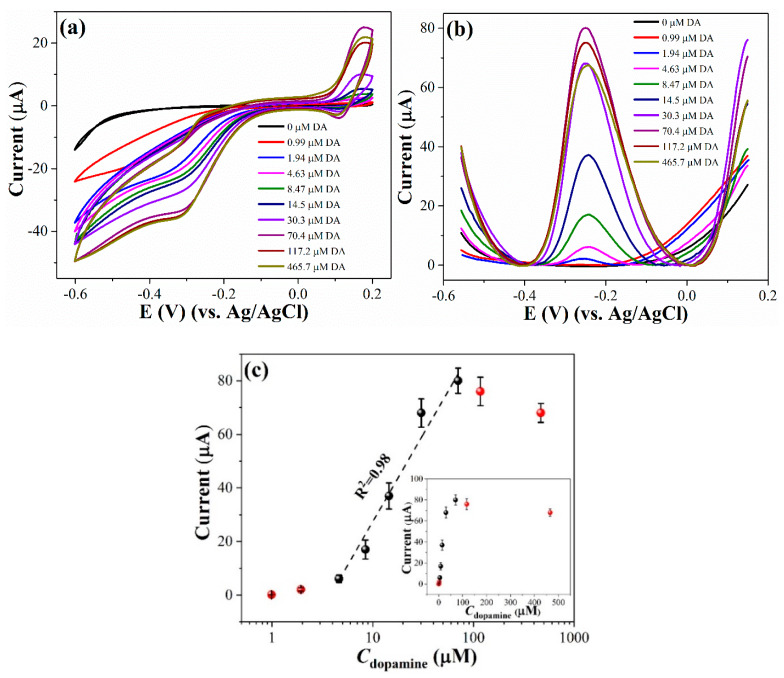
(**a**) Cyclic voltammetry curves and (**b**) square wave voltammetry (SWV) current responses of split aptamer-based electrochemical sandwich assays for the detection of different dopamine concentrations; (**c**) dependence of the electrochemical current on dopamine concentration.

High background signals are a common problem associated with conventional amperometric aptamer sensors where a redox tag is attached to the distal end of the aptamer. To this end, an electrochemical sandwich assay, obtained by splitting a full aptamer into two fragments, is regarded as an available platform to suppress the background signal and to enhance the potential change on the gate electrode induced by analyte binding for potentiometric transducer [31,35]. In the present work, two-fragmented aptamer strands were utilized for detecting the small molecule neurotransmitter, dopamine. One strand (aptamer1) was covalently attached on the surface of a gold macroelectrode via a thiol–gold bond and the other aptamer fragment (aptamer2) was modified with the redox moiety methylene blue. In the absence of dopamine, no charge transfer was observed between the aptamer and the gold electrode, even in Tris buffer containing 0.5 μM of the methylene blue-modified fragment (Figure 1a, black curve), indicating that aptamer2 freely floated in the buffer solution and no binding event between the two split aptamer parts occurred. The addition of the analyte dopamine, with a concentration of approximately 1 μM, resulted in the occurrence of a distinct Faraday current (Figure 1a, red curve), presumably due to the formation of sandwich assembly, which brought the redox tag in close proximity to the electrode surface. The peak current of around −200 mV to −300 mV (vs. Ag/AgCl) after target addition was generated by the redox conversion of methylene blue, while the anodic signal (around 100 mV) was presumably caused by the oxidation and reduction of dopamine directly. Both signals depended strongly on the concentration of dopamine. A sensitive square wave voltammetry (SWV) technique was utilized to quantitatively record the response of the split aptamer-based electrochemical sandwich assay (SAESA) to different concentrations of dopamine molecules. The operation frequency of the SWV measurements was firstly optimized to obtain the highest possible current response of the SAESA to the analyte (Figure S2) and a final frequency of 70 Hz was chosen as the operation frequency for all following SWV measurements. A negligible background current was observed in the absence of dopamine (Figure 1b, black curve), while there was an obvious increase in the Faraday current with rising dopamine concentrations registered at the peak potential of around −250 mV (vs. Ag/AgCl) in the presence of the aptamer2 fragment. A control experiment was performed to characterize the response of the aptamer1-modified gold electrode to different concentrations of dopamine without the presence of aptamer2, where no current response was observed (Figure S3), indicating that the specific binding between the split aptamer and dopamine causes the current response. The corresponding Faraday currents were plotted versus the concentration of dopamine to obtain a calibration curve for the amperometric detection scheme (Figure 1c). A steep and linear current increase was observed for the concentration range between 5 μM and 70 μM (limit of linearity for the semi-logarithmic presentation). A further increase in the target concentration to 117.2 μM resulted in a decrease in the corresponding peak current. This observation can most likely be attributed to the formation of polydopamine at high monomer concentrations (Figure 1c) which may, on the one hand, have interfered with the formation of aptamer/analyte sandwich structures, and on the other hand, hindered the charge transfer between the redox probes and the electrode by fouling the electrode surface [36]. The detection limit of this amperometric sandwich assay for the detection of dopamine was 1 μM, determined according to international union of pure and applied chemistry (IUPAC) instructions. 

**Figure 2:**
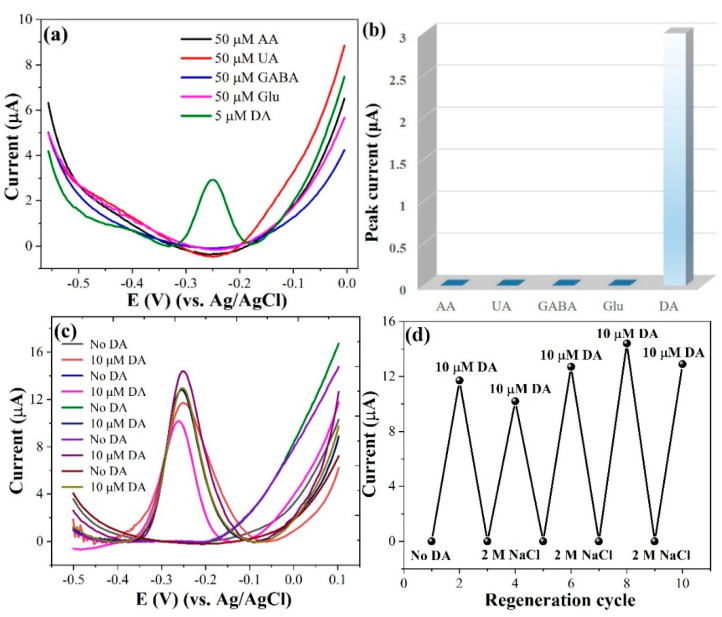
(**a**) SWV current responses and (**b**) column comparison graph of the corresponding current of the split aptamer-based electrochemical sandwich assay for the detection of dopamine (DA) and its analogues: ascorbic acid (AA), uric acid (UA), gamma-aminobutyric acid (GABA), and glutamate (Glu); (**c**) SWV curves and (**d**) the corresponding current responses showing the regeneration of aptasensors after repeated rinsing of the amperometric aptasensor with a 2 M NaCl solution.

Furthermore, the selectivity of the amperometric SAESA was evaluated by comparing the current response of the aptasensor for dopamine relative to its analogues, which are in part also important neurotransmitters or interfering sample species with redox characteristics similar to dopamine (Figure 2a,b). A distinct current peak evolved with the presence of 5 μM dopamine, while no peaks were generated for ascorbic acid (AA), uric acid (UA), gamma-aminobutyric acid (GABA), and glutamic acid (Glu), even at concentrations (50 μM) 10-fold higher than that of dopamine (5 μM), which demonstrated the high selectivity of the SAESA for dopamine. Regeneration tests were carried out to characterize the reusability of the aptasensor by consecutively soaking it into the regeneration agent containing 2 M NaCl (Figure 2c,d) which interrupted the aptamer–dopamine complex without damaging the integrity of the surface tethered aptamer1 and permitted the recovery of the dopamine-binding aptamer quadruplex structure as soon as the regeneration solution was replaced by the dopamine/aptamer2/Tris buffer [37]. After the regeneration treatment, the observed current signal was around zero, which is same as the background signal without the administration of dopamine, confirming the removal of aptamer2 carrying the redox probe (Figure 2c). The extremely low background signal will lead to a large on-to-off ratio of the current signal, which make the SAESA a promising alternative for detecting low concentrations of dopamine. The subsequent addition of 10 μM dopamine exhibited similar responses as for the original SAESA, indicating that the covalently bonded aptamer1 remained functional. The SAESA responses exhibited ~116.7% recovery even after experiencing three detection cycles, demonstrating the excellent reusability of the SAESA for dopamine detection. However, the clinical concentrations of dopamine are very diverse, depending on the considered location in a biological system, and can be as small as a few fM in single adrenal chromaffin cells [38], 1 nM in human serum [39], or 10 nM in the brain [40]. Therefore, a detection limit of 1 μM and a concentration range of approximately one order of magnitude strongly limits the applicability of the amperometric aptasensor. To boost the detection performance, the described sensor was extended by an interdigitated organic electrochemical transistor (iOECT) as an amplifying transducer.

**Figure 3:**
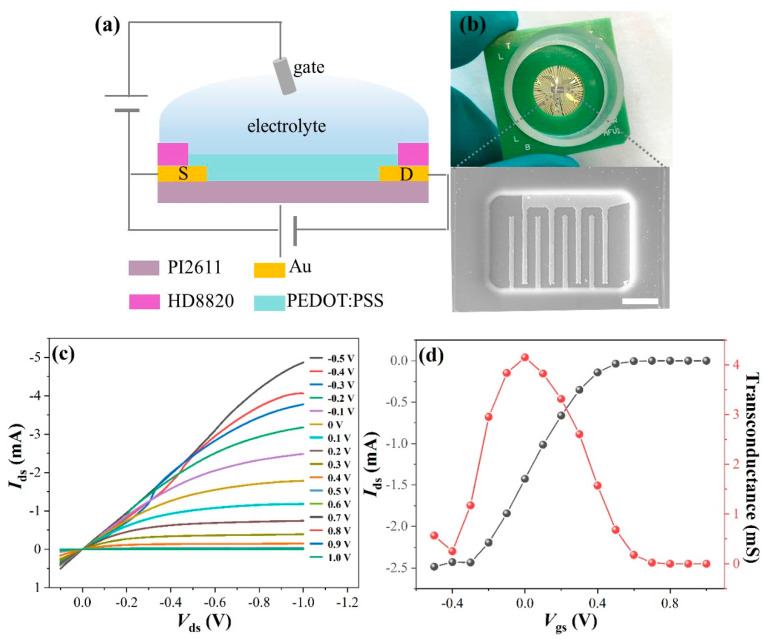
(**a**) Schematic configuration of organic electrochemical transistors (OECTs) embedded in a flexible substrate polyimide. (**b**) Digital photograph of the encapsulated flexible device. The SEM image shows a magnified individual transistor. The scale bar is 10 μm. (**c**) The output characteristics of the flexible OECTs obtained by using a Ag/AgCl pellet as a gate electrode at *V*_gs_ varying from −0.5 V to 1.0 V with a step width of 0.1 V. (**d**) The extracted transfer characteristics from (**c**) at *V*_ds_ = −0.5 V.

In our previous work on flexible iOECTs, we proved their high flexibility and electrical performance. In this work, flexible iOECTs were utilized as a transducer [24,26] to detect dopamine by means of the same split aptamer-modified Au electrode, as described above, but now operated as a gate (Figure 3a). The channel area (a polymer specifically between both electrodes) of the selected iOECT was 30 μm × 24 μm (Figure 3b), which is comparable to the size of an individual electrogenic cell [33]. The general output characteristics of the flexible iOECTs with a drain–source bias (*V*_ds_) varying from −1.0 V to 0.2 V, and a gate–source bias (*V*_gs_) in the range of −0.5 V and 1.0 V, were measured by using a standard Ag/AgCl pellet as the gate electrode (Figure 3c). Depending on the applied organic channel materials, OECTs can work in accumulation and depletion modes [41]. The output drain–source current (*I*_ds_) increased with *V*_ds_ following Ohm’s law [42] until the saturation was reached (Figure 3c) while it decreased with increasing *V*_gs_, especially for a *V*_gs_ higher 0 V. This observation can be attributed to the depletion mode characteristics of PEDOT:PSS-based OECTs. Once a positive gate bias is applied at the gate bias, anions from electrolytes accumulated around the gate electrode and cations inversely penetrated into the PEDOT:PSS channel, which compensated the pendant sulfonate anions on the PSS, resulting in a de-doping of PEDOT. As a consequence, the hole density in the channel decreases, accompanied by the decline of the drain–source current. The transconductance, which represents the capability to convert changes in the gate potential into variations of the source–drain channel current [22], was determined (Figure 3c). The normalization of the maximum transconductance with the applied *V*_ds_ was 8 mS/V (Figure 3d), which is superior to other reported flexible OECTs [43,44]. Additionally, the output and transfer characteristics of the flexible iOECTs were measured using the Au electrode as a gate electrode (Figure S4) exhibiting a similar response to that using a Ag/AgCl pellet. As a consequence, the high transconductance endows the flexible iOECTs with outstanding amplification capability [24], which is crucial to the following application of dopamine detection.

**Figure 4:**
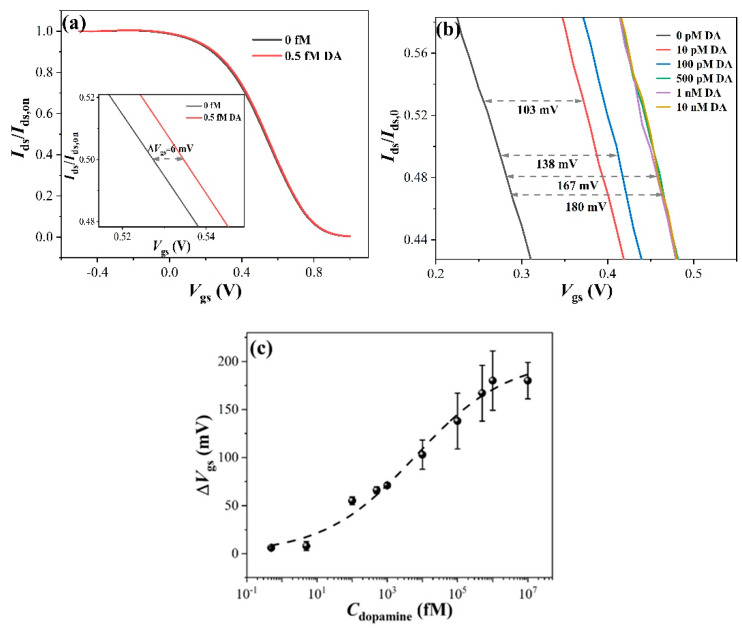
Transfer characteristics of flexible interdigitated organic electrochemical transistors (iOECTs) versus gate voltage at *V*_ds_ = −50 mV measured in 10 mM Tris buffer containing 0.5 μM aptamer2 before and after the administration of different concentrations of the dopamine target. The target incubation time was 10 min. The concentration of dopamine in the Tris buffer was (**a**) 0.5 fM and (**b**) varying from 0 pM to 10 nM. (**c**) The logarithmic dependence between the shift in gate potentials (Δ*V*_gs_) and target concentrations.

To permit a direct comparison of the sensitivity between the abovementioned amperometric aptasensor and the flexible iOECT transducer for the detection of dopamine, the aptamer1-modified Au electrode was used for both direct amperometric detection and utilization as a gate electrode for the PEDOT:PSS transistor (Figure S5). The organic transistor demonstrated its performance by recording the potential change in the gate electrode as a function of analyte concentration (Figure 4). To compensate for possible instabilities in the electrical performance of iOECTs during long-term measurements (Figure S6), at least four different iOECTs were used for the detection of dopamine with the same concentration. In addition, the gate electrode was fixed in a micromanipulator of a probe station and the iOECT chamber was kept dry during the incubation of the target on the gate. Instead, a sacrificial encapsulated iOECT chip with completely the same configuration as the one used for dopamine detection was employed for the incubation of the target to reduce the contact of the latter with the electrolyte [24]. The transfer characteristics of the flexible iOECTs were measured before and after the addition of different concentrations of dopamine targets in a 0.5 μM aptamer2/Tris buffer solution at *V*_ds_ = −50 mV. To evaluate the response of the iOECT-based transducer and exclude the influence of variable initial source–drain currents caused by the electrical instability of the PEDOT:PSS film in the electrolyte during the measurements of different target concentrations, the relative current change *I*_ds_/*I*_ds,on_ = 0.5 was determined before and after incubation with dopamine (Figure 4). Interestingly, the addition of dopamine to the aptamer-modified gate electrode at a concentration as low as 0.5 fM caused a shift in the transfer curve of the iOECT of approx. 6 mV to a higher gate potential (Figure 4a), indicating an extremely low detection limit of the flexible polymer transducer. A significant change in the gate potential of 103 mV was observed for a dopamine concentration of 10 pM (Figure 4b) and the potential shift increased with further rising analyte concentrations. This shift to higher gate potentials can be attributed to the introduction of negatively charged aptamer2 strands to the modified Au electrode during the dopamine binding events [45], which thus reduced the surface potential of the gate electrode [24]. The resulting changes of gate potential can be considered as offset voltage, which is caused by the potential drop at the gate/electrolyte and electrolyte/organic channel layer. To maintain the original effective source/gate bias applied on the organic channel material, a higher gate voltage was required to compensate for the potential drop caused by the binding of dopamine and aptamer2, resulting in the shift of the transfer curve towards higher potentials. 

To permit a comparison of both transducers, the calibration curve was determined as well, by plotting the shift of the gate potential versus target concentration (Figure 4c and Table S1). A typical S-shaped curve was observed for the potentiometric sensor, which can be divided into three regimes. At a target concentration lower than 5 fM (*C*_dopamine_ ≤ 5 fM), the gate potential exhibited weak dependence on the target concentration. The rise in the target concentration from 5 fM to 1 nM resulted in an almost linear increase in the potential shift. This regime was followed by the third region for high analyte concentration (*C*_dopamine_ > 1 nM), where the potential change saturated. The detection limits and the detection range of the flexible iOECT sensor are summarized in Table 1 for comparison with other previously reported electrochemical sensors for dopamine detection. It is noteworthy that an ultralow detection limit was obtained for the iOECT-based transducer, which was nine orders of magnitude lower than that of the corresponding amperometric aptasensor (1 μM) and superior to all other previously reported electrochemical sensors [30,46]. The extraordinarily low detection limit of the flexible polymer transducer can be attributed to its high transconductance in combination with a relatively low number of receptors bound to the surface of the gate electrode. In addition, the analyte binding occurs in association with the aptamer2 strand, which further enhances the sensor signal. In our previous study, a signal amplification of four orders of magnitude in comparison to an amperometric transducer for the binding of ATP to a full aptamer, and a significant shift in *V*_gs_ by the immobilization of ssDNA molecules on the gate electrode, were observed [21]. Additionally, the obtained dissociation constant (*K*_D_) of the split aptamer sequences with dopamine targets for the amperometric and potentiometric transducers using a Langmuir equation [47] were 5 µM and 7 pM, respectively (Supplementary Materials). These results, in combination with the high signal amplification observed here, suggest that the incubation of the split aptamer2 strand together with the analyte is advantageous over full aptamer receptors for OECT sensors, where only the analyte itself is bound to the surface-tethered receptor molecules.

Furthermore, the novel iOECT-based transducer exhibited an extremely wide detection range for dopamine concentrations, varying from 5 fM to 1 nM. Since both amperometric and potentiometric transducers are using the same gold electrode, they can be operated together to broaden the detection range of the combined sensor (5 fM to 1 nM potentiometric + 5 μM to 50 μM amperometric), and thus extend its field of applications for the detection of dopamine in plasma, cells, and urine. 

**Figure 5:**
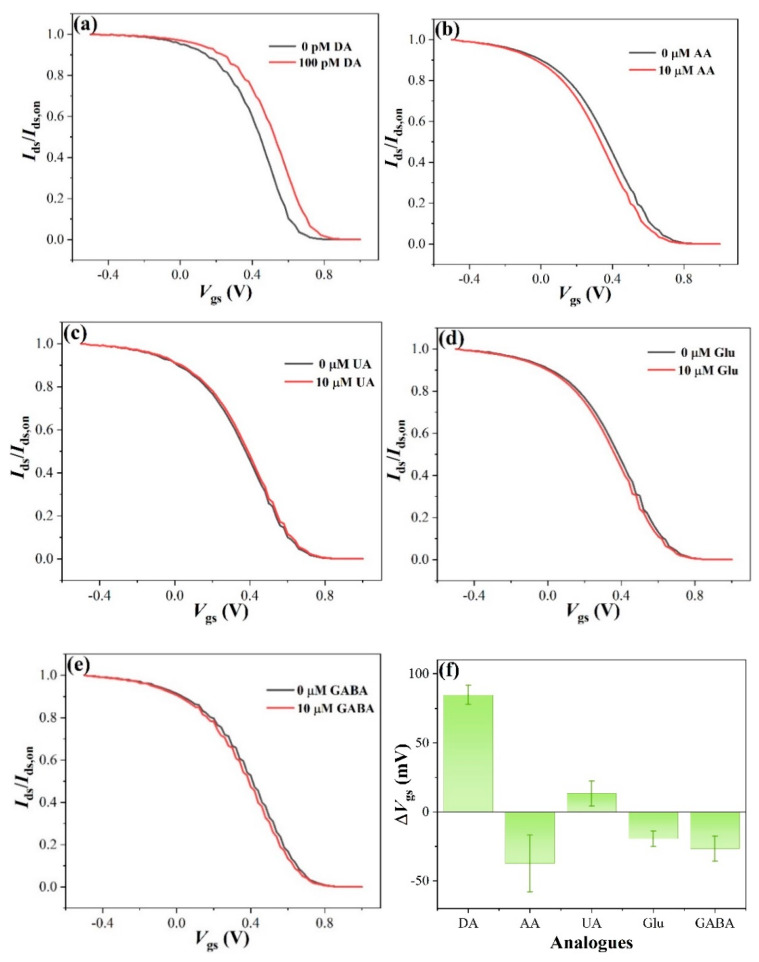
Transfer characteristics of the flexible iOECTs with (**a**) 100 pM dopamine (DA), 10 μM (**b**) ascorbic acid (AA), (**c**) uric acid (UA), (**d**) glutamate (Glu), and (**e**) Gamma-aminobutyric acid (GABA). (**f**) Column comparison graph of the shifts in gate potential obtained from (**a**–**e**).

In addition to the sensitivity and detection range, the selectivity was evaluated as a crucial parameter of the aptasensor performance. The response of the transfer characteristics of the iOECTs to selected interfering substances such as ascorbic acid (AA), uric acid (UA), glutamate (Glu), and gamma-aminobutyric acid (GABA) was characterized for this purpose (Figure 5). Five independent iOECT devices were used for the detection of each target molecule to eliminate device-related variations. The changes in gate potentials caused by the unspecific binding of dopamine analogues were quite small (less than 15 mV) or even shifted towards the reverse direction in comparison with that of the specific dopamine binding (approx. 85 mV) (Figure 5f) although the concentration (10 μM) of the former was 100 times higher than that of the target analyte (100 pM). This observation confirms firstly that the high selectivity of the split aptamer was also conserved for the iOECT transducer, and secondly that neither the presence of dopamine nor aptamer2 in the analyte affects the integrity of the iOECT device.

## 4. Conclusions

In this work, flexible interdigitated organic electrochemical transistors (OECTs) were utilized for detecting the small molecule neurotransmitter dopamine. The background signal of the amperometric sensor significantly decreased by introducing split ssDNA aptamers with a resultant detection limit of 1 μM. This sandwich assay, formed by two aptamer fragments plus the analyte, exhibited high selectivity for the detection of dopamine among other neurotransmitters and excellent reusability. In comparison, the iOECT transducer exhibited a significantly improved sensitivity for dopamine detection with an ultralow detection limit, which was nine orders of magnitude lower than the corresponding amperometric transducer and lower than all other previously reported electrochemical sensors. The high sensitivity of the flexible iOECT-based aptasensor can be attributed to its intrinsic amplification capability. Furthermore, the high selectivity of the aptamer receptor could be conserved for the flexible iOECT transducer, where dopamine was distinctly distinguished from other important neurotransmitters. The high sensitivity, selectivity, regeneration capability, and conformability of the flexible OECT-based aptasensor pave the way for medical applications where excellent sensor performance is required without limitations from motions or the shape of the target object.

## Figures and Tables

**Table 1:** Comparison of different types of biosensors for dopamine detection.

Biosensors	Methods	Detection Limit	Linear Calibration Range	Selectivity	Ref.
Electrochemically preanodized clay-modified electrode	Square wave voltammetry	2.7 nM	3	No	[48]
Graphene-modified electrode	Differential pulse voltammetry	5 µM	2 (5–175 µM)	Yes	[7]
PDMS/glass microchip	Hydrodynamic voltammogram catecholamines	2 µM	1 (20 to 100 μM)	No	[4]
MWCNTs, quercetin, and Nafion-modified glassy carbon electrode	Amperometric detection (current density versus time)	4.72 µM	2 (5–500 μM)	Yes	[46]
Au nanoparticles decorated polypyrrole/reduced graphene oxide hybride sheets	Differential pulse voltammetry	18.2 pM	4 (0.1 nM to 5 μM)	No	[2]
Polyvinylpyrrolidone/graphene modified glassy carbon electrode	Amperometric detection (current versus time)	0.2 nM	7 (0.0005–1130 μM)	Yes	[8]
Aptamer–field-effect transistors	Transfer characteristics	1 fM	4 (1 fM–10 pM)	Yes	[6]
Split-aptamer sensor	Square wave voltammetry	1 µM	1 (5 µM–50 µM)	Yes	Present work
Split aptamer-based OECT sensor	Transfer characteristics	0.5 fM	5 (5 fM–1 nM)	Yes	Present work

## Supplementary Materials

The following is available online at https://www.mdpi.com/1996-1944/13/11/2577/s1, Figure S1: Optimization of the incubation time of amperometric aptasensor with target molecule dopamine (50 μM), Figure S2: The optimization of operation frequency for SWV measurements at a concentration of dopamine of 30 µM, Figure S3: The SWV response of amperometric aptasensor to different concentrations of dopamine without the addition of the aptamer2 molecule, Figure S4: The output and transfer characteristics of iOECTs by using a gold electrode as the gate electrode, Figure S5: The photograph of aptamer-modified gold electrode integrated with flexible iOECTs, Figure S6: The variation of source–drain current with time measured in 10 mM Tris buffer at applied *V*_ds_ = −0.05 V and *V*_gs_ = 0.35 V, Table S1: The gate potential (*V*_gs_) and corresponding potential change ∆*V*_gs_ (mV) at *I*_ds_/*I*_ds,on_ = 0.5 of transfer characteristics of an iOECT chip at different dopamine concentrations.
