# Supplementary material for: Label-Free Split Aptamer Sensor for Femtomolar Detection of Dopamine by Means of Flexible Organic Electrochemical Transistors
Source: Materials (Basel). 2020 Jun 5;13(11):2577. doi: 10.3390/ma13112577 (PMC7321560; doi:10.3390/ma13112577)
Supplement: Supplementary file 1 [file materials-13-02577-s001.pdf]

materials

Supplemental Materials

Label-Free Split Aptamer Sensor for Femtomolar Detection of Dopamine by Means of Flexible Organic Electrochemical Transistors

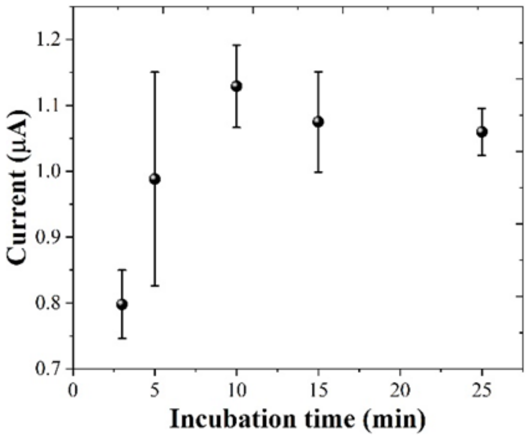

Figure S1. Optimization of the incubation time of amperometric aptasensor with target molecule dopamine (50 μM).

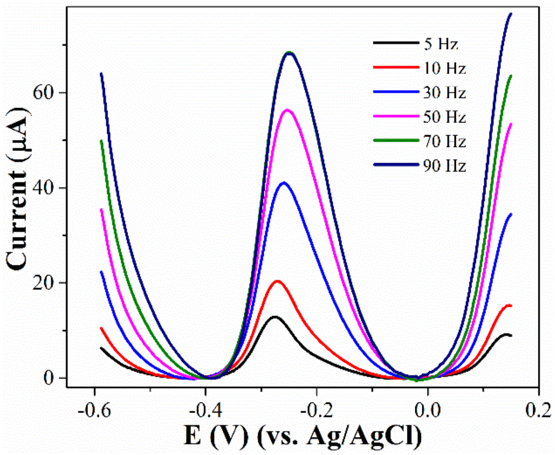

Figure S2. The optimization of operation frequency for SWV measurements at the concentration of dopamine of 30 μM.

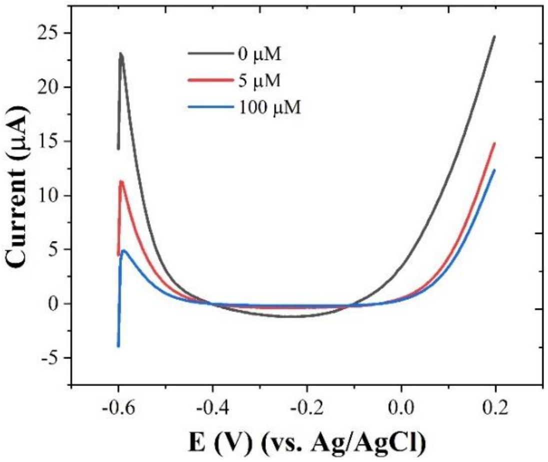

Figure S3. The SWV response of amperometric aptasensor to different concentration of dopamine without the addition of aptamer 2 molecule.

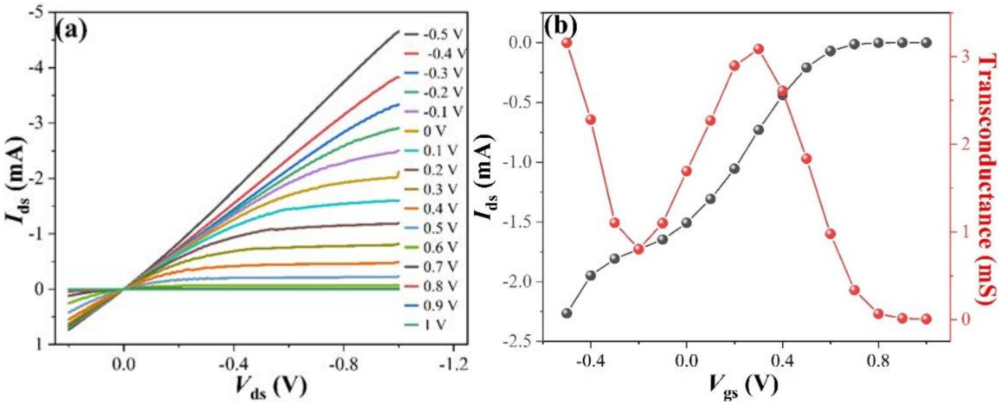

Figure S4. The output and transfer characteristics of iOECTs by using a gold electrode as the gate electrode.

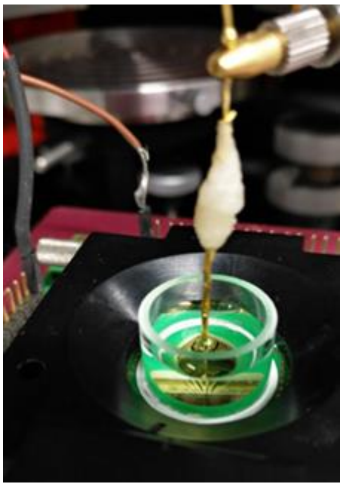

Figure S5. The photograph of aptamer-modified gold electrode integrated with flexible iOECTs.

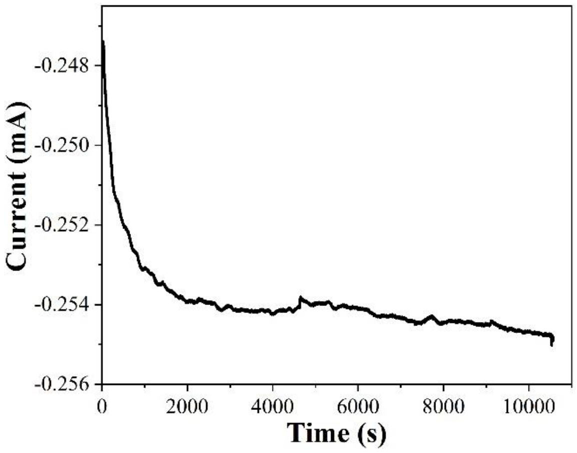

Figure S6. The variation of source-drain current with time measured in 10 mM tris buffer at applied $V_{ds} = -0.05$ V and $V_{gs} = 0.35$ V.

Table S1. The gate potential (V_{gs}) and corresponding potential change ΔV_{gs} (mV) at $I_{ds}/I_{ds,on} = 0.5$ of transfer characteristics of an iOECT chip at different dopamine concentrations.

**Device**	**Gate Potential (V_{gs})/ΔV_{gs} (mV) at $I_{ds}/I_{ds,on}$ = 0.5**										
	**DA: 0**	**0.5 fM**	**5 fM**	**100 fM**	**500 fM**	**1 pM**	**10 pM**	**100 pM**	**500 pM**	**1nM**	**10 nM**
OECT01	528	534/6	-	-	-	-	-	-	-	-	-
OECT02	517	514/−3	-	-	-	-	-	-	-	-	-
OECT03	457	462/5	-	-	-	-	-	-	-	-	-
OECT04	453	459/4	-	-	-	-	-	-	-	-	-
OECT05	496	-	504/8		-	-	-	-	-	-	-
OECT06	498	-	514/16	-	-	-	-	-	-	-	-
OECT07	505	-	523/18	-	-	-	-	-	-	-	-
OECT08	485	-	496/11	-	-	-	-	-	-	-	-
OECT09	483	-		538/55	549/ 64	554/71	-	-	-	-	-
OECT10	454	-	-	517/63	527/ 73	530/76	-	-	-	-	-
OECT11	442	-	-	502/60	513/ 71	515/73	-	-	-	-	-
OECT12	272	-	-	-	-	-	385/ 103	410/ 138	449/ 167	452/ 180	452/ 180
OECT13	289	-	-	-	-	-	372/83	386/97	419/ 130	426/ 137	443/ 154
OECT14	316	-	-	-	-	-	389/73	398/82	426/ 110	435/ 120	459/ 143

The dissociation constant (K_D) of the split aptamer sequences with dopamine target can be obtained by fitting the dependence curves of the electrochemical current on dopamine concentration using a Langmuir equation [1], which is described as:

$$I_{\text{target}} = I_0 + \left(\frac{C_{\text{target}}(I_{\max} - I_0)}{C_{\text{target}} + \mathrm{K_D}}\right)$$

where I_{target} and I_0 are the signal current with and without the presence of dopamine, respectively. I_{max} is the maximum current, C_{target} is the target concentration. It is noteworthy, that we determined two calibration curves for exactly the same chemical equilibrium system, which was read out by two independent transducer systems delivering two very different K_D values, being K_D = 5 µM and K_D = 7

pM for the amperometric and the potentiometric transducer, respectively. This finding emphasizes that the measured K_D differs from the theoretical K_D since the actual measured K_D value depends not only on the binding affinity of the receptor to its ligand but also on the sensitivity of the transducer, which converts the binding event into a detectable signal for this kind of detection system.

Reference

1. Idili, A.; Parolo, C.; Ortega, G.; Plaxco, K.W. Calibration-free measurement of phenylalanine levels in blood using an electrochemical aptamer-based sensor suitable for point-of-care applications. *ACS Sens.* **2019,** *4*, 3227–3233.
